# Aspirin-induced nuclear translocation of NF*κ*B and apoptosis in colorectal cancer is independent of p53 status and DNA mismatch repair proficiency

**DOI:** 10.1038/sj.bjc.6602455

**Published:** 2005-03-15

**Authors:** F V N Din, L A Stark, M G Dunlop

**Affiliations:** 1 Colon Cancer Genetics Group, Department of Oncology, Division of Clinical and Molecular Medicine and MRC Human Genetics Unit, Western General Hospital, University of Edinburgh, Crewe Rd, Edinburgh EH4 2XU, Scotland

**Keywords:** NF-*κ*B, p53, mismatch repair, NSAIDs, colorectal cancer

## Abstract

Substantial evidence indicates nonsteroidal anti-inflammatory drugs (NSAIDs) protect against colorectal cancer (CRC). However, the molecular basis for this anti-tumour activity has not been fully elucidated. We previously reported that aspirin induces signal-specific I*κ*B*α* degradation followed by NF*κ*B nuclear translocation in CRC cells, and that this mechanism contributes substantially to aspirin-induced apoptosis. We have also reported the relative specificity of this aspirin-induced NF*κ*B-dependent apoptotic effect for CRC cells, in comparison to other cancer cell types. It is now important to establish whether there is heterogeneity within CRC, with respect to the effects of aspirin on the NF*κ*B pathway and apoptosis. p53 signalling and DNA mismatch repair (MMR) are known to be deranged in CRC and have been reported as potential molecular targets for the anti-tumour activity of NSAIDs. Furthermore, both p53 and MMR dysfunction have been shown to confer resistance to chemotherapeutic agents. Here, we set out to determine the p53 and hMLH1 dependency of the effects of aspirin on NF*κ*B signalling and apoptosis in CRC. We specifically compared the effects of aspirin treatment on cell viability, apoptosis and NF*κ*B signalling in an HCT-116 CRC cell line with the p53 gene homozygously disrupted (HCT-116^p53−/−^) and an HCT-116 cell line rendered MMR proficient by chromosomal transfer (HCT-116^+ch3^), to the parental HCT-116 CRC cell line. We found that aspirin treatment induced apoptosis following I*κ*B*α* degradation, NF*κ*B nuclear translocation and repression of NF*κ*B-driven transcription, irrespective of p53 and DNA MMR status. These findings are relevant for design of both novel chemopreventative agents and chemoprevention trials in CRC.

Ingestion of nonsteroidal anti-inflammatory drugs (NSAIDs) has been shown to be associated with a 40–50% reduction in relative risk of colorectal cancer (CRC) (Thun *et al*, 1993). There is much research effort focussed on the molecular mechanisms involved in NSAID anti-tumour activity. Such understanding would inform the rational design of novel agents both for chemoprevention and therapy.

We previously reported the pivotal involvement of the transcription factor NF*κ*B (p50–p65) in aspirin-mediated apoptosis in CRC cells (Stark *et al*, 2001). NF*κ*B is sequestered in the cytoplasm, bound to a member of the I*κ*B family of inhibitory proteins. Stimulation by one of a range of signals results in phosphorylation and ubiquitination of I*κ*B followed by proteosome-mediated degradation. Dissociation from I*κ*B results in NF*κ*B nuclear translocation and transcriptional regulation of numerous target genes. NF*κ*B has been shown to have both pro- and anti-apoptotic effects (Barkett and Gilmore, 1999). Such disparate effects are due to differences in stimuli, NF*κ*B composition, cell type and distinct *κ*B binding specificities of individual complexes resulting in diverse target gene specificity (Epinat and Gilmore, 1999). We have previously shown that aspirin-mediated apoptosis in CRC cells involves I*κ*B*α* degradation and NF*κ*B nuclear translocation (Stark *et al*, 2001). We also demonstrated that aspirin-induced I*κ*B*α* degradation was required for apoptosis because cells constitutively expressing super-repressor I*κ*B*α* were resistant to both aspirin-induced NF*κ*B nuclear translocation and apoptosis. More recently, we have reported the relative specificity of this NF*κ*B-dependent apoptotic effect of aspirin for CRC cells, when compared to other cancer cell types (Din *et al*, 2004). However, it is also important to determine whether there is heterogeneity within CRC, with respect to the effects of aspirin on the NF*κ*B pathway and apoptosis.

It is now well established that genomic instability, by increasing mutational load, promotes neoplastic progression in CRC. The p53 tumour suppressor gene is involved in cell cycle control, apoptosis and maintenance of genomic stability and is frequently mutated in colorectal tumours, heralding malignant transformation (Baker *et al*, 1989, 1990; Honma *et al*, 2000). Another important contributor to genomic instability is defective DNA mismatch repair (MMR) resulting in microsatellite instability (MSI) (Veigl *et al*, 1998). MSI is the hallmark of tumours arising in hereditary nonpolyposis colorectal cancer (HNPCC) and is also found in 15% of sporadic CRCs (Boland *et al*, 1998; Brown *et al*, 1998). The majority of MSI tumours in familial cases are due to germline mutations in hMLH1, hMLH2 and hMSH6 genes, and to hMLH1 promoter hypermethylation in sporadic cancers (Kuismanen *et al*, 2000). p53 dysfunction and DNA MMR deficiency are almost wholly mutually exclusive (Cottu *et al*, 1996; Samowitz *et al*, 2001). p53 signalling and DNA MMR have been identified as potential molecular targets for NSAIDs (Ruschoff *et al*, 1998; Shao *et al*, 2000; Goel *et al*, 2003), suggesting that the anti-tumour effect may, in part, involve countering the effects of genetic instability in CRC. Such genetic aberrations in tumours have also been shown to be involved in determining response to chemotherapeutic agents (O'Connor *et al*, 1997; Weller, 1998; Ribic *et al*, 2003).

In light of the importance of p53 and MMR in CRC and that genomic instability can influence response to chemotherapeutics, we set out to determine the p53 and hMLH1 dependency of the effects of aspirin on NF*κ*B signalling. Our findings suggest that the NF*κ*B-apoptotic response to aspirin occurs irrespective of p53 status and MMR defects and shed further light on the chemopreventive action of NSAIDs.

## MATERIALS AND METHODS

### Cell line culture and treatment

The CRC cell line HCT-116 (genotype p53 (+/+), hMLH1 (−)), is available from the American Type Culture Collection (ATCC). The HCT-116 cell line has a hemizygous mutation in hMLH1 resulting in a truncated, nonfunctional protein. The HCT-116 subline where hMLH1 expression is restored by chromosome 3 transfer (HCT116^+ch3^, genotype p53 (+/+), hMLH1 (+)) was a gift from Professor CR Boland and these cells are competent in DNA MMR (Koi *et al*, 1994). The p53 null HCT-116 subline (HCT-116^p53−/−^) was created by targeted homologous recombination (Bunz *et al*, 1999) and was a gift from Professor B Vogelstein. All three cell lines were grown in McCoy's 5A media and the HCT-116^+ch3^ cell line was grown under selection with 0.4 mg ml^−1^ geneticin. All media were supplemented with 10% foetal calf serum (FCS) and 1% penicillin/streptomycin (media supplied by Gibco BRL, Paisley, UK) and cells were grown as monolayers (37°C in 5% CO_2_). Cells were plated (1 × 10^6^ cells/50 ml flask) and grown until 60–70% confluent. Aspirin (Sigma, St Louis, USA) was prepared as a 0.5 M stock solution in distilled water (final pH 7.0). Prior to treatment for 16 h with aspirin (1, 3, 5 and 10 mM) or carrier control (at same concentrations as aspirin), the growth medium was replaced with the respective low serum (0.5% FCS) medium.

### Cell viability and determination of apoptosis

After aspirin treatment, adherent cells were harvested and viable cell number determined by counting with a haemocytometer. Cell surface phosphatidylserine is a marker for apoptosis and was detected via its interaction with annexin V using the Annexin V-FITC apoptosis detection kit (Oncogene Research Products, Cambridge, MA, USA), as per the manufacturer's instructions. Briefly, the media from the flask of adherent cells was transferred to a conical tube on ice to harvest any floating cells. Cells were then washed with 2 ml of PBS, which was also added to the tube to collect any cells dislodged during washing. Cells were incubated with 1 ml of trypsin : versene (volume per volume) just until the cells detached and then resuspended in the conical tube containing the media with the floating and washed cells. Cells were counted using a haemocytometer and resuspended in cold 1 × binding buffer to approximately 1 × 10^6^ cells ml^−1^. Media binding reagent (10*μ*l) was added to 0.5 ml of the cell suspension, which was incubated with 1.25 *μ*l of annexin V-FITC for 15 min at room temperature in the dark. Annexin V was then removed by centrifugation at 1000 × **g** for 5 min and the cells were resuspended in 0.5 ml of cold 1 × binding buffer and placed on ice. The counting was carried out using a haemocytometer (two counting grids) in duplicate and this was carried out immediately following staining of cells as apoptosis is an ongoing process and the FITC signal may be lost after an hour.

### Western blotting

After aspirin treatment, cells were washed with PBS, centrifuged (1200 r.p.m. for 10 min) and cell pellets were resuspended in lysis buffer (50 mM NCl, 10 mM HEPES, 500 mM sucrose, 1 mM EDTA, 0.5 mM. spermidine, 0.15 mM spermine, 0.2% Triton X-100, Complete Protease Inhibitor Cocktail (Roche Diagnostics, Manheim, Germany), 100 mM Pefabloc (Roche Diagnostics, Manheim, Germany)). The cell suspension was centrifuged (6000 r.p.m. for 15 min, 4°C) and the supernatant used as cytoplasmic extract. Protein content was measured by the method of Bradford (BioRad, Hercules, CA, USA).

Cytoplasmic proteins (30 *μ*g) were separated on a 10% SDS–PAGE gel, transferred to a polyvinylidine difluoride membrane (BioRad) and blocked in 4% non-fat dry milk solution with 0.3% Tween20 (Sigma). Membranes were probed with a sheep polyclonal I*κ*B*α* antibody (a gift from Professor R Hay, University of St Andrews, UK) and then with monoclonal antibody to Cu/Zn SOD (The Binding Site, Birmingham, UK) as a control for protein loading. Chemiluminescence was used to visualise the antigen–antibody complexes (Amersham ECL Reagents, Little Chalfont, UK).

### Electrophoretic mobility shift assays

Electrophoretic mobility shift assays were carried out by incubating nuclear extracts from untreated cells (6 *μ*g) with binding reaction mix (1 × binding buffer (50 mM KCl, 20 mM HEPES, 5% glycerol, 1 mM EDTA, 1 mM DTT), 1 *μ*g BSA, 1 *μ*g poly dI-dC, 25 fmol radioactively labelled oligo-DNA in a final volume of 20 *μ*l) for 30 min prior to analyses on a 4% native polyacrylamide gel. Double-stranded oligonucleotides for NF*κ*B were obtained from Santa Cruz.

### Immunofluorescence analysis

Cells grown to 60–70% confluence on glass coverslips were treated with carrier or 10 mM aspirin for 16 h (in respective 0.5% FCS medium). After treatment, cells were washed with PBS, fixed with acetone : methanol (volume per volume) (−20°C, 10 min) and blocked in 10% nonimmune donkey serum (Sigma) for 1 h. Rabbit polyclonal antibody to NF*κ*B p65 (Santa Cruz) was applied for 1 h followed by incubation with FITC-conjugated donkey anti-rabbit IgG for 1 h. The nuclei were stained with DAPI and the coverslips mounted with Vectashield (Vector Laboratories, Burlingame, California, USA).

### Transfections and reporter assays

For transient transfection experiments, 50 ml flasks of cells were grown to subconfluency then transfected with 6 *μ*g of luciferase reporter plasmid and 3 *μ*g of *β*-galactosidase control plasmid, using Lipofectamine as described by the manufacturer's instructions (Gibco BRL). Following transfection, cells were grown in low serum (0.5% FCS) medium then treated with aspirin (0–10 mM) for 16 h. Luciferase activity was measured in cell extracts using a luciferase reporter assay kit (Promega) and read using a luminometer. Transfection efficiency and cell viability were monitored by cotransfection with a CMV-*β*-galactosidase reporter plasmid and *β*-galactosidase activity was quantified with an assay kit (Promega), as per the manufacturer's instructions. Relative luciferase activity was calculated as unit of luciferase activity per unit of *β*-galactosidase activity.

## RESULTS

### Basal expression of I*κ*B*α* and p65 proteins in CRC cell lines is independent of p53 and hMLH1 expression

**Figure 1:**
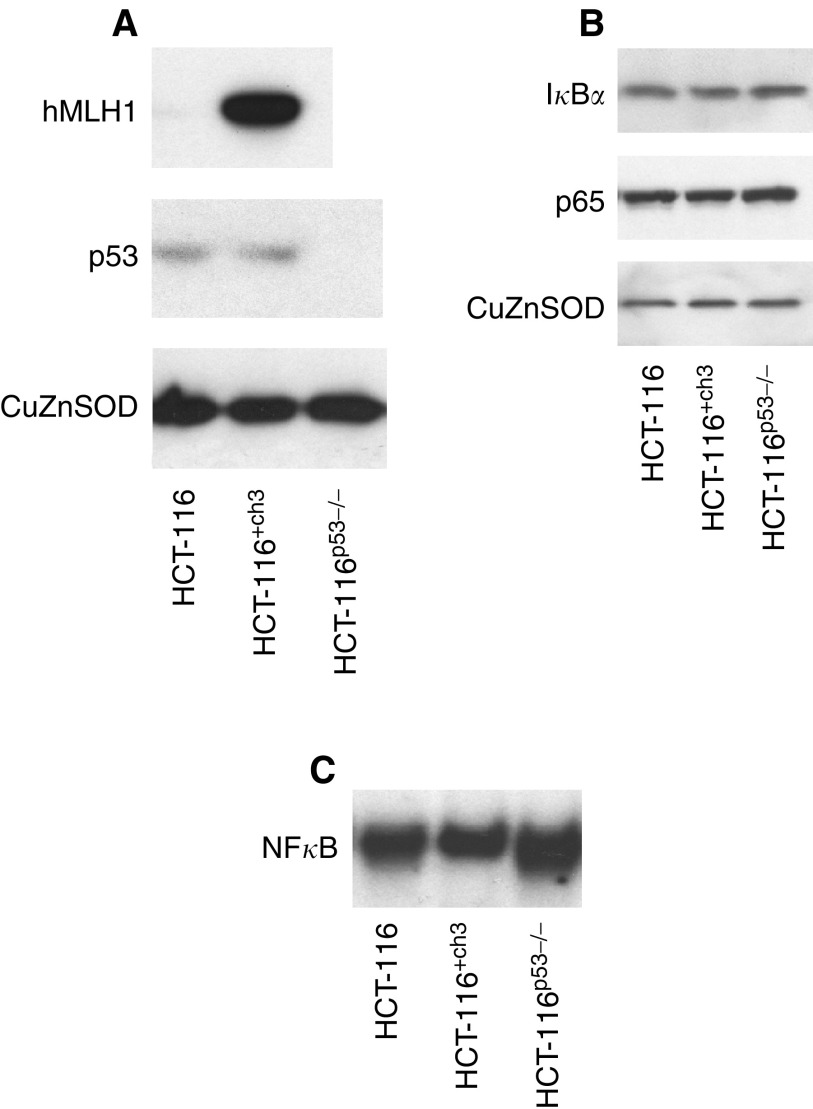
Basal expression of p53, hMLH1, I*κ*B*α* and p65 in untreated colorectal cancer cell lines. Cytoplasmic extracts were made from untreated cells and Western blots probed with p53 and hMLH1 antibodies to confirm the expression profile of the cell lines (**A**). Cytoplasmic extracts were probed with sheep polyclonal I*κ*B*α* and rabbit polyclonal p65 antibodies to examine the basal expression of proteins in the cell lines (**B**). The Western blots shown are representative of at least three independent experiments and Cu/Zn SOD was used as a control for protein loading. Electrophoretic mobility shift assay using NF*κ*B consensus oligonucleotide, performed on nuclear extracts of untreated cells, demonstrated basal NF*κ*B DNA binding activity (**C**).

To investigate the relationship between p53 and MMR status and the NF*κ*B-dependent apoptotic response to aspirin, we used the following three CRC cell lines: parental HCT-116 cells (wild-type p53, hMLH1 deficient), HCT-116^+ch3^ (wild-type p53, hMLH1 proficient) and HCT-116^p53−/−^ (p53 null, hMLH1 deficient). The presence of hMLH1 in the HCT-116^+ch3^ cell line and the absence of p53 in the HCT-116^p53−/−^ cell line was verified by immunoblot analysis (Figure 1A). Firstly, we considered whether p53 and hMLH1 mutation status might affect basal levels of I*κ*B*α* and p65 proteins. Using immunoblot analysis of cytoplasmic extracts, we found there were no significant differences in expression of I*κ*B*α* or p65, or their relative levels (data not shown), between the three CRC cell lines (Figure 1B). These results indicate that changes in MMR mutation status and p53 expression do not affect the cytoplasmic pool of either protein available for stimulation. We next considered whether basal levels of NF*κ*B DNA binding were affected by changes in p53 or MMR status. Electrophoretic mobility shift assays (EMSAs) performed on nuclear extracts of untreated cells showed basal NF*κ*B DNA binding in the three CRC cell lines (Figure 1C). The differences in basal levels of NF*κ*B DNA binding complexes in the HCT-116^+ch3^ and HCT-116^p53−/−^ cell lines compared to the parental HCT-116 cell line were marginal (densitometry data not shown).

### Aspirin induces apoptosis in CRC cell lines independent of p53 and MMR status

**Figure 2:**
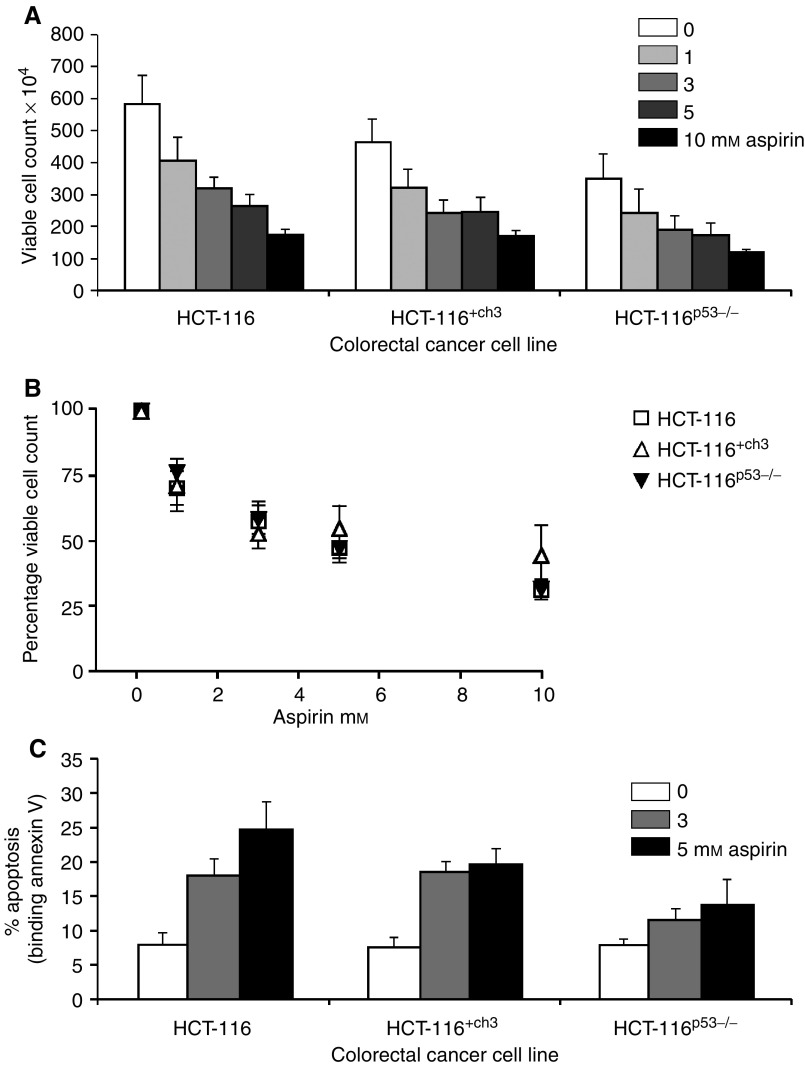
Effect of aspirin on cell viability and apoptosis in colorectal cancer (CRC) cell lines. Aspirin treatment (0–10 mM) for 16 h induces a concentration-dependent decrease in viable cell number (determined by haemocytometric counts) in all CRC cell lines (**A**). The decrease in cell viability is proportionate at each concentration increment indicating a similar pattern of response following aspirin treatment in each cell line (**B**). Annexin V binding assay used to determine that all CRC cell lines undergo apoptosis after aspirin treatment (0–5 mM) (**C**). The graphs represent three independent experiments and the bars on the graphs are standard error bars.

To determine whether p53 and MMR status affects the aspirin-mediated reduction in cell viability we have previously observed, we compared the effects of aspirin treatment on the viability of HCT-116^+ch3^ and HCT-116^p53−/−^ cell lines to the parental HCT-116 cell line. In triplicate dose–response experiments, all CRC cell lines were treated for 16 h with aspirin (1, 3, 5 and 10 mM) and viable cell number determined by haemocytometric counts. Aspirin treatment resulted in a concentration-dependent decrease in the number of viable cells in all three CRC cell lines (Figure 2A). Furthermore, the cell lines showed proportionate decreases in cell viability at each concentration increment, indicating a similar sensitivity to aspirin, irrespective of p53 or MMR status (Figure 2B). The IC_50_ values were calculated from the growth curves of the aspirin-treated CRC cell lines (Table 1) and there were no significant differences in levels of cell death with each genetic background.

It is well established that aspirin induces apoptosis in CRC cell lines (Piazza *et al*, 1995; Qiao *et al*, 1998; Stark *et al*, 2001; Din *et al*, 2004). We next confirmed that the reduction in viable cell number in each of the cell lines was indeed due to apoptosis for all three genotypes, and not simply a growth inhibitory effect. Following aspirin treatment, cells were stained with Annexin V, which binds phosphatidylserine residues that are externalised during apoptosis and thus serves as a marker for programmed cell death. Consistent with the reduction in cell viability, we found that aspirin induced a concentration-dependent increase in the proportion of apoptotic cells in all three CRC cell lines (Figure 2C). Furthermore, there was no significant difference in apoptotic response between any of the HCT-116, HCT-116^+ch3^ and HCT-116^p53−/−^ cell lines. In addition there was good correlation between the IC_50_ value and apoptotic response; the lower the IC_50_ value, indicating greater sensitivity at a lower aspirin concentration, the greater the fold increase in apoptosis (*r*=−0.98). These data suggest that aspirin-mediated apoptosis in CRC cells is independent of p53 and MMR status.

### Aspirin induces I*κ*B*α* degradation and NF*κ*B nuclear translocation in CRC cell lines irrespective of p53 and MMR status

**Figure 3:**
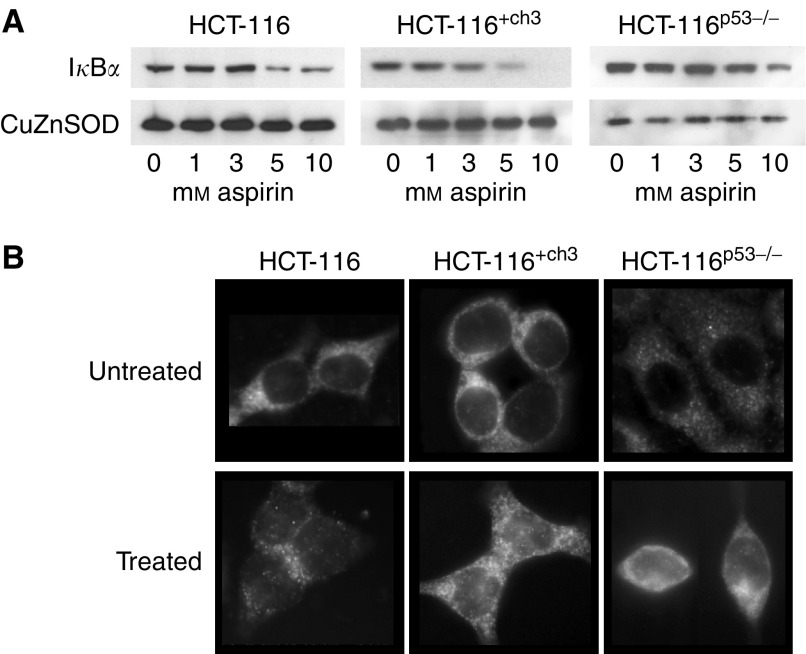
Aspirin induces I*κ*B*α* degradation and nuclear translocation of p65 in colorectal cancer (CRC) lines. Following aspirin treatment, cytoplasmic extracts were made from untreated and treated cells and probed with sheep polyclonal I*κ*B*α* antibody. Western blot analysis shows that aspirin treatment (0–10 mM) for 16 h induces I*κ*B*α* degradation in a concentration-dependent manner in the HCT-116, HCT116^+ch3^ and HCT-116^p53−/−^ CRC cell lines (**A**). The Western blot shown is representative of at least three independent experiments and Cu/Zn SOD was used as a control for protein loading. Micrographs (63 ×) of immunocytochemically stained cells show that aspirin treatment (10 mM) for 16 h induces nuclear accumulation of p65 in the HCT-116, HCT116^+ch3^ and HCT-116^p53−/−^ CRC cell lines (**B**).

Having shown previously that p65 nuclear translocation is a critical event in effecting the apoptotic response to aspirin (Stark *et al*, 2001), we next studied the effect of aspirin on NF*κ*B signalling in each cell line. Since I*κ*B*α* sequesters NF*κ*B in the cytoplasm, we first investigated the effect of aspirin treatment on the cytoplasmic levels of I*κ*B*α* in each cell line using immunoblot analysis. We found that aspirin treatment induced degradation of I*κ*B*α* in a similar concentration-dependent manner in each cell line genotype (Figure 3A). We next determined whether the I*κ*B*α* degradation was accompanied by NF*κ*B nuclear translocation in the CRC cell lines. Immunofluorescence analysis showed that following 16 h aspirin exposure, p65 translocated from the cytoplasm to the nucleus in each cell line, irrespective of p53 and MMR status (Figure 3B). These findings indicate that aspirin-induced apoptosis, due to modulation of the NF*κ*B pathway, occurs irrespective of derangements in p53 signalling and the MMR system.

### Aspirin-mediated repression of NF*κ*B-driven reporter activity is unrelated to p53 and MMR status

**Figure 4:**
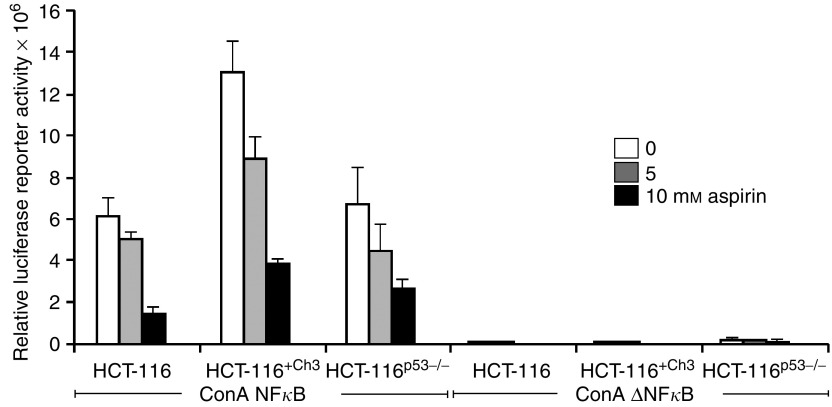
Aspirin induces repression of NF*κ*B-driven transcription in colorectal cancer (CRC) lines. CRC cells were transfected with the ConA NF*κ*B dependent luciferase reporter construct, containing three *κ*B binding sites, or the equivalent plasmid with *κ*B consensus sites deleted (ConA Δ*κ*B). All cells were cotransfected with the control CMV-*β*-galactosidase plasmid. Following 16 h treatment with 0–10 mM aspirin, luciferase and *β*-galactosidase assays were performed on cell lysates and relative luciferase activity calculated. The graphs represent three independent experiments and the bars on the graphs are standard error bars.

We have previously demonstrated aspirin-induced nuclear translocation of NF*κ*B results in repression of NF*κ*B transcriptional activity in CRC cell lines (Stark *et al*, 2000). This was recently substantiated by the finding that NF*κ*B induced by some cytotoxic stimuli acts as an active repressor of anti-apoptotic gene expression (Campbell *et al*, 2004). The effect of aspirin on NF*κ*B-driven transcription was investigated to determine whether p53 or MMR mutation status affects the ability of aspirin to induce NF*κ*B transcriptional repression in CRC cells. The cell lines were transiently transfected with the 3enhancer-ConA NF*κ*B-dependent luciferase reporter construct in which transcription of the firefly luciferase gene is driven by three *κ*B binding sites (Roff *et al*, 1996). A reporter plasmid with deleted *κ*B sites served as control. Following transfection, cells were exposed to aspirin for 16 h. There was a substantial decrease in the basal levels of NF*κ*B-driven reporter activity following aspirin exposure and this was observed in a concentration-dependent manner, irrespective of p53 or MMR status (Figure 4). These findings show that aspirin-induced repression of NF*κ*B transcriptional activity, and hence downstream regulation of target genes, is independent of p53 signalling and DNA MMR.

## DISCUSSION

We previously reported that the effects of aspirin on NF*κ*B signalling are a centrally important mechanism of aspirin-mediated apoptosis in CRC cells (Stark *et al*, 2001; Din *et al*, 2004). This work considerably extends these previous observations since we have shown that the effect of aspirin on apoptosis and NF*κ*B signalling is independent of p53 and DNA MMR status. Furthermore, we show that aspirin induces nuclear translocation of p65 that is associated with repression of *κ*B-driven transcription, again independent of p53 or MMR status.

It is well established that aspirin has a chemopreventive effect in CRC, but the mechanism of action has not been fully characterised. It is apparent that inhibition of the cyclooxygenase-2 enzyme (COX-2) plays a part in the anti-tumour effect of NSAIDs in CRC (Marnett and DuBois, 2002). However, it is also clear that there are other important mechanisms involved since the growth inhibitory and apoptotic effects of NSAIDs occur in CRC cell lines that do not express COX-2 (Hanif *et al*, 1996; Elder *et al*, 1997). Furthermore, NSAIDs that lack COX-2 activity are growth inhibitory and also induce apoptosis in CRC cells (Piazza *et al*, 1995, 1997; Elder *et al*, 1997). It has also been shown that NSAID concentrations required for growth inhibition differ from those for COX-2 enzyme inhibition (Charalambous and O'Brien, 1996; Hanif *et al*, 1996). Taken together, such evidence indicates that while COX-2 inhibition is important, there are other essential mechanism(s) of action. p53 function and DNA MMR have been proposed as potential targets responsible for the anti-tumorigenic properties of NSAIDs (Ruschoff *et al*, 1998; Shao *et al*, 2000).

The p53 signalling pathway is central to regulating cell growth and death, and stabilisation of p53 by mutation is a key event occurring late in colorectal tumorigenesis (Baker *et al*, 1990). Several studies have shown an association between p53 mutation status and sensitivity to chemotherapeutic drugs in colorectal and other cancers (O'Connor *et al*, 1997; Weller, 1998). The p53 pathway has been postulated as a potential target since NSAIDs have been shown to alter levels of p53 (Goldberg *et al*, 1996; Kralj *et al*, 2001). Furthermore, there is evidence for regulatory interdependence between p53 and NF*κ*B involving competition for common coactivators (Wu and Lozano, 1994; Webster and Perkins, 1999). Indeed, wild-type p53 has been shown to suppress constitutive NF*κ*B activity and lead to apoptosis (Shao *et al*, 2000), suggesting that tumours expressing wild-type p53 may be more susceptible to aspirin-induced apoptosis.

Our previous work indicated that CRC cell lines expressing wild-type p53 were not more sensitive to aspirin-induced apoptosis mediated by NF*κ*B signalling, but there are differences other than p53 status between the cell lines studied (Din *et al*, 2004). In this study, we specifically investigated the effects of p53 on the NF*κ*B-induced apoptotic response by use of HCT-116 cells with the p53 gene homozygously disrupted by targeted homologous recombination (Bunz *et al*, 1999). Using this approach, we have shown that aspirin-induced apoptosis is independent of p53 in CRC cells. Furthermore, p53 does not appear to play a role in aspirin-induced effects on NF*κ*B signalling or on the repression of NF*κ*B transcriptional activity. These findings are important in terms of chemoprevention since p53 mutant CRCs have been shown to differ in behaviour from those expressing wild-type p53, with respect to response to chemotherapeutic agents and prognosis.

Defective DNA MMR is characteristic of HNPCC and around 15% of sporadic CRCs also exhibit genetic instability, mainly due to epigenetic silencing of hMLH1 but also to somatic MMR gene defects (Herman *et al*, 1998). The DNA MMR system has been implicated as a potential pathway for modulation that may contribute to NSAID anti-tumour activity (Ruschoff *et al*, 1998; Goel *et al*, 2003). Our previous work suggested that MMR proficient cells may be more sensitive to aspirin-induced apoptosis since the MMR-deficient cell lines had greater IC_50_ values than MMR-proficient cell lines (Din *et al*, 2004). Hence, we examined whether MMR status influenced NF*κ*B-dependent aspirin-induced apoptosis, by comparing the MMR-deficient HCT-116 to its proficient counterpart HCT116^+ch3^. We observed a dose-dependent increase in apoptosis after treatment with aspirin following I*κ*B*α* degradation, NF*κ*B nuclear translocation and repression of NF*κ*B-driven transcription in both cell lines. The parental HCT-116 cells, which are MMR deficient, did appear to have a marginally lower IC_50_ value and show a greater fold increase in apoptosis when compared to the MMR-proficient cell line, but this was not significant (Table 1). Although we did not detect any significant differences in the NF*κ*B-dependent apoptotic response to aspirin attributable to MMR status, long-term *in vitro* aspirin exposure has been shown to select for microsatellite stability in colorectal and gastric cancer cell lines (Ruschoff *et al*, 1998; Yamamoto *et al*, 1999). It has also recently been shown that aspirin treatment increased MMR protein expression and apoptosis in CRC cell lines (Goel *et al*, 2003). However, we found no evidence that MMR status influences the NF*κ*B-dependent apoptotic response, suggesting that the MMR system is not the predominant pathway responsible for NSAID anti-tumour activity.

This work consolidates our previous findings that aspirin-induced apoptosis occurs after I*κ*B degradation, NF*κ*B nuclear translocation and repression of NF*κ*B-driven transcription. The results presented here shed further light on the complex mechanisms by which NSAIDs induce cell death in CRC. Elucidation of the mechanism lies in defining the relative contribution of putative targets to aspirin's anti-tumour activity. We found no evidence for the involvement of p53 or DNA MMR on inducing the NF*κ*B pathway, nor on the ensuing apoptotic response. Genomic instability due to p53 or MMR dysfunction has been to shown to be associated with resistance to chemotherapeutic agents. Hence, these findings may inform rational design of novel therapeutics. In addition, since aspirin effects on NF*κ*B and apoptosis occur in cancers arising from different genetic backgrounds, these findings are clinically relevant when considering design of chemoprevention trials, both in genetically predisposed individuals with defective MMR and in low and moderate risk populations, since p53 mutational events are important during development and progression of colorectal neoplasia.

## Figures and Tables

**Table 1:** IC_50_ values for colorectal cancer cell lines

**CRC cell line**	**IC_50_**	**Fold increase apoptosis**	
		**3 mM**	**5 mM**
HCT-116	2.8	2.3	3.1
HCT-116^+ch3^	3.1	2.4	2.6
HCT-116^p53−/−^	4.3	1.5	1.8

Cells were treated with aspirin (1,3,5,10 mM) for 16 h and cell numbers determined as described in Materials and Methods. There is an inverse correlation between IC_50_ values and the fold increase in apoptosis at both 3 and 5 mM (*r*=−0.93 and −0.98). Results are mean of at least three different experiments.
